# The Artelon CMC spacer compared with tendon interposition arthroplasty: A randomized, controlled, multicenter study of 109 patients with osteoarthritis followed for 1 year

**DOI:** 10.3109/17453671003635835

**Published:** 2010-04-06

**Authors:** Anders Nilsson, Monica Wiig, Håkan Alnehill, Magnus Berggren, Sten Björnum, Mats Geijer, Philippe Kopylov, Christer Sollerman

**Affiliations:** ^1^Department of Hand Surgery, Sahlgrenska University Hospital, Göteborg; ^2^Department of Hand Surgery, Uppsala University Hospital, Uppsala; ^3^Department of Hand Surgery, Örebro University Hospital, Örebro; ^4^Department of Hand and Plastic Surgery, Linköping University Hospital, Linköping; ^5^Gothenburg Medical Center, Göteborg; ^6^Department of Radiology, Sahlgrenska University Hospital, Göteborg; ^7^Department of Orthopedics, Lund University Hospital, LundSweden

## Abstract

**Background and purpose** The Artelon CMC spacer is designed for surgical treatment of osteoarthritis (OA) in the carpometacarpal joint of the thumb (CMC-I). Good results using this degradable device were previously presented in a pilot study. We now present results from a larger randomized, controlled, multicenter study.

**Patients and methods** 109 patients (94 females) with a mean age of 60 (42–83) years, suffering from painful CMC OA, were included in the study at 7 centers in Sweden. The patients were randomized to Artelon CMC spacer (test, n = 72) or tendon arthroplasty (control, n = 37) at a ratio of 2:1. Perceived pain was recorded on a visual analog scale (VAS) before treatment and after 3, 6, and 12 months, when measuring maximal tripod pinch strength (primary outcome measure). In addition, range of motion, radiographic findings, and functional testing were recorded pre- and postoperatively.

**Results** Swelling and pain were more common in the test group and 6 implants were removed because of such symptoms. 5 of these patients did not receive antibiotics preoperatively according to the study protocol. In a per-protocol analysis, i.e. patients without signs of concomitant OA in the scaphoid-trapezium-trapezoid (STT) joint and those in the test group who received antibiotics, the mean difference in tripod pinch strength increase, adjusted for baseline, was 1.4 kg in favor of the test group (not statistically significant). Statistically significant pain relief was achieved in both groups, with perceived pain gradually decreasing during the follow-up period. In the intention-to-treat analysis but not in the per-protocol analysis, significantly better pain relief (VAS) was obtained in the control group. Patient-perceived disability evaluated by the DASH questionnaire improved in both groups.

**Interpretation** The Artelon CMC spacer did not show superior results compared to tendon interposition arthroplasty. Proper use of preoperative antibiotics and a thorough patient selection appear to be important for the results.

## Introduction

Surgical treatment of carpometacarpal (CMC) osteoarthritis (OA) includes several different techniques, which have been compared in previous studies (Gervis 1949, Wolock et al. 1989, Amadio and De Silva 1990, Kuschner and Lane 1996, Gibbons et al. 1999, Mureau et al. 2001). Soft tissue interposition, with or without ligament reconstruction (Burton and Pellegrini 1986, Froimson 1987, Weilby 1988, Sigfusson and Lundborg 1991), and silicone elastomer arthroplasty (Swanson et al. 1981) result in good pain relief, but some long-term studies have shown disabling weakness of the pinch grip (Tomaino et al. 1995, Hartigan et al. 2001).

Endoprostheses have been used, either as a total joint replacement with preserved os trapezium or as an implant replacing os trapezium (Regnard 2006). Both implant designs are associated with subluxation, material fatigue, and occurrence of wear debris causing adverse tissue reactions (Swanson et al. 1981, Sollerman et al. 1993, Bezwada et al. 2002, Perez-Ubeda et al. 2003).

Nilsson et al. (2005) presented a T-shaped CMC device made of a degradable polyurethane urea (Artelon) and evaluated the results in a pilot study. The device has two modes of action: it stabilizes the CMC joint by augmentation of the joint capsule and it resurfaces the distal part of the trapezial bone. The selection of a degradable biomaterial for the CMC spacer device was based on a biological approach to support the local tissue repair. The purpose of the device was to provide a scaffold for tissue ingrowth and to prevent impingement between the bones of the CMC joint. In the pilot study, this implant showed superior results compared to tendon interposition arthroplasty (Nilsson et al. 2005).

A larger randomized, controlled, multicenter study has now been performed to further evaluate the benefits of this method. Patients included in the study were randomized to the CMC joint spacer or tendon interposition arthroplasty surgery at a ratio of 2:1. Here we present the 1-year results from this study.

## Patients and methods

A randomized, controlled, and observer-blinded multicenter study was started at 7 Swedish hospitals. The study plan was reviewed and approved by the local university ethics committees in Göteborg (S 301-01; T 356-03), örebro (890/01), Uppsala (Ups 01-365), Lund (LU 544-01), Linköping (LIU 02-402), and Stockholm (KI 01-354) according to the Declaration of Helsinki of 1975, as revised in 2000.

**Figure 1.**
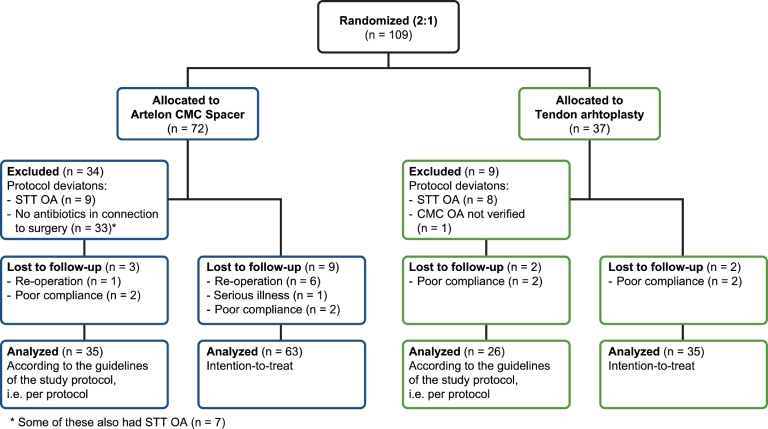
Flow chart of patients with CMC-I osteoarthritis (OA).

The study included 109 consecutive patients (111 thumbs) with painful and radiographically verified OA (Eaton stage 1–3) in the CMC joint (Eaton and Glickel 1987). Exclusion criteria were OA in the scaphoid-trapezium-trapezoid (STT) joint, serious illness, an ongoing infection, or malignancy within the previous 10 years. After giving informed consent, the patients were randomized to the Artelon CMC spacer (test group) or tendon interposition arthroplasty (control group) at a ratio of 2:1 (Figure 1), according to a randomization list and by using closed envelopes. The test group had 72 patients (61 females) and the control group had 37 patients (33 females). Their mean ages were 59 (42–77) years and 61 (45–83) years, respectively. The patients were operated on between March 2002 and August 2004. The numbers of thumbs operated on at the 7 hospitals varied between 7 and 23. The dominant hand, in most cases the right hand, was operated in 31 and 22 patients in the test and control group, respectively (42% and 59%). 6 patients were withdrawn from the test group before the 1-year follow-up due to reoperation and 1 patient because of serious illness. 2 patients in each group did not come to the 1-year follow-up visit (Figure 1).

The pre- and postoperative examinations included both subjective and objective tests, and they were performed by blinded observers, i.e. the observers carrying out the follow-up investigations were not informed about which surgical procedure the individual patient had undergone. Clinical instability, swelling, bleeding, erythema, and infections were recorded at all visits.

Pain was registered according to a visual analog scale (VAS; 0 = no pain, 10 = unbearable pain), when measuring maximal tripod and key pinch. Pinch strength—both tripod (primary outcome measure) and key pinch—was measured with a pinch gauge (North Coast Medical Inc., Morgan Hill, CA). Transverse volar grip strength was measured with a dynamometer (Jamar; Sammons Preston Inc., Bolingbrook, IL). Measurements were made with the patient sitting in a chair with the elbow resting on a table. Furthermore, radial and palmar thumb abduction were measured with a goniometer and recorded between the first and second metacarpal. Patient-perceived disability was evaluated using the DASH questionnaire (Disability of the Arm, Shoulder, and Hand) (Turchin et al. 1998, Atroshi et al. 2000). The scores from all items were used to calculate a total score ranging from 0 (no disability) to 100 (most severe disability). The patients' satisfaction with treatment and their subjective experiences were recorded on a scale from 1 (not at all) to 5 (very satisfied/very good).

Radiography was performed pre- and postoperatively with dedicated postero-anterior (PA) and lateral views of the thumb and first metacarpal, also including the trapezium and scaphoid. Additional oblique or Eaton projections were obtained when needed. All preoperative and 1-year radiographs were evaluated by an independent observer. The joint space width of the CMC-I joint and the degree of subluxation of the first metacarpal were measured with a magnifying loupe with a sub-millimeter graded caliper on films, and with measuring tools (software) on digital images. The degree of OA in the CMC-I joint was graded according to Eaton (North and Eaton 1983, Eaton and Glickel 1987).

### Surgical technique

**Figure 2.**
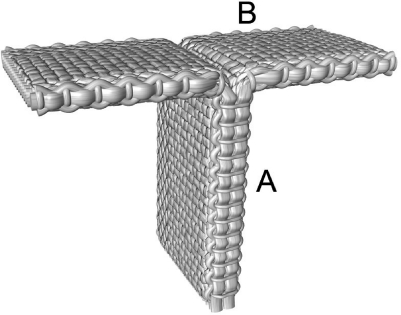
The Artelon CMC spacer device with the vertical spacer (A) and the 2 horizontal wings (B).

The Artelon material is manufactured by Artimplant AB, Sweden, and has been described in earlier reports (Gisselfält et al. 2002, Liljensten et al. 2002, Gretzer et al. 2003). The Artelon CMC spacer device is T-shaped with a vertical spacer serving as an interposition in the CMC joint, and 2 horizontal wings augmenting the dorsal joint capsule in order to prevent dorso-radial migration of the proximal metacarpal (Figure 2).

Implantation of the spacer was performed through a dorsal approach, exposing the CMC joint with a proximal-based capsular incision (Nilsson et al. 2005). Approximately 2 mm of the distal surface of the trapezium and any osteophytes were excised with a chisel. The device was inserted with the vertical part into the joint space and the wings anchored to cancellous bone on each side of the joint. Fixation was achieved with non-resorbable osteosutures.

In the control group, the trapezium was resected in all cases but the tendon interposition was performed with different techniques according to the established routine at each center. The abductor pollicis longus (APL) tendon was used for interposition in 22 cases (Sigfusson and Lundborg 1991), the extensor carpi radialis longus (ECRL) tendon in 6 cases (Necking and Eiken 1986), and the Burton procedure in 9 cases (Burton and Pellegrini 1986). The postoperative treatment was the same in all patients with 5–6 weeks of plaster fixation followed by a mobilization program.

### Statistics

The patients were randomly allocated to test group and control group at a ratio of 2:1. In order to detect a clinically relevant difference of 1 kg in tripod pinch strength between the groups after 1 year at a significance level of 0.05 and with a power of 80%, at least 56 subjects were needed in the test group and 28 subjects in the control group, assuming the SD in both groups to be 0.6 kg. To compensate for any dropouts, 72 subjects were randomized to the test group and 36 to the control group. Change over time within groups was analyzed with Student's t-test for paired analyses. For comparison between groups in change from baseline to 1-year follow-up, analysis of variance (ANCOVA) was used with adjustment for baseline values. Adjusted mean differences between groups are given with 95% confidence intervals (CIs). In 2 patients, both thumbs were treated. In 1 of these patients, the thumbs were randomized to each of the two groups. The values from both of these thumbs are accounted for in the analysis. This would give a slightly conservative test. The other patient was randomized to the test treatment in both thumbs and the mean of these 2 measurements was calculated when included in the analyses, i.e. the statistical analyses were based on patient as the unit. Significance tests were two-tailed and were conducted at the 5% significance level.

## Results

### Intention-to-treat analysis, i.e. all patients included in the study

**Figure 3.**
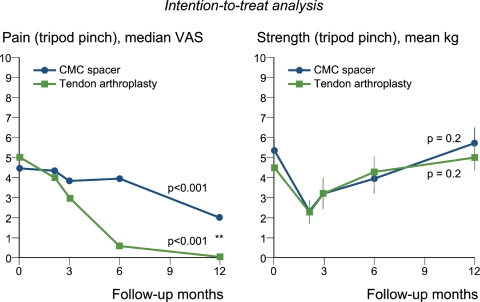
The intention-to-treat analysis (i.e. involving all patients included in the study) of pain according to VAS (left panel) and strength (right panel) at maximal loading in tripod pinch (pinch gauge) before treatment and during 1 year after surgery, for patients treated with the Artelon CMC spacer (n = 65) or trapezium excision and tendon interposition (n = 35). Dots and error lines show median/mean values and confidence intervals, and the p-values are for change up to 1 year. **p < 0.01 for difference between groups.

Tripod pinch strength (primary outcome measure) decreased immediately after surgery, but both groups had regained their pinch and grip strength after 1 year without any statistically significant difference between the groups (Table and Figure 3, right panel). Perceived pain decreased significantly during the follow-up period in both groups according to the VAS measurements (Figure 3, left panel), with significantly more pain relief in the control group (Table).

The evaluation of 107 available preoperative radiographs (96%) by an independent observer revealed no radiographic OA in 1 case, OA in the CMC-I joint of Eaton stage 1 in 8 cases (7%), stage 2 in 60 cases (56%), stage 3 in 29 cases (27%), and stage 4 in 9 cases (8%). Except for the latter 9 cases with Eaton stage 4, STT OA was seen in 8 further patients who were graded as being Eaton stage 1–2. In total, the evaluation revealed 17 cases (16%) with osteoarthritic changes in the STT joint: 9 in the spacer group and 8 in the control group.

1-year radiographs were available for 57 patients (90%) in the spacer group. 4 possible complications were noted: 2 cases with a high degree of subluxation, 1 case of severe osteoporosis, and 1 patient with possible osteonecrosis of the trapezium. Postoperative radiography of all patients in the control group did not reveal any abnormalities other than the expected absence of the trapezium.

At the 1-year follow-up, mild to moderate swelling was noted in 21 thumbs (32%) in the test group and in 1 thumb (3%) in the control group. Complications in the test group during follow-up included removal of 6 CMC spacers mainly because of pain. In 2 of these cases, infection was suspected but bacterial cultures were negative. Histological evaluation of the explanted spacers showed no signs of inflammation located in close proximity to the Artelon material. In 2 cases, however, inflammatory cells could be detected in the surrounding soft tissue and near and inside the suture used for fixation, respectively. Other complications in the spacer group were presence of pain in 7 other patients at the 6- or 12-month follow-ups. No complications related to the treatment were reported in the control group.

### Per-protocol analysis, i.e. patients who followed all details in the study protocol

Deviations from the intended study protocol were found in 40% of the cases (Figure 1) and thus a per-protocol analysis was also performed excluding the following patients: 17 patients with STT OA—9 in the spacer group and 8 in the control group—had been included and 1 patient in the control group without radiographically verified CMC-I OA, when the radiographs were analyzed by the independent observer. According to the protocol, 1 dose of preoperative antibiotics should have been administered in the spacer group. However, 46% (33/72) of the patients did not receive this.

**Figure 4.**
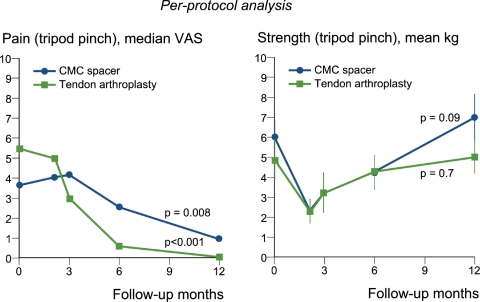
The per-protocol analysis (i.e. involving patients who followed all details in the study protocol) of pain according to VAS (left panel) and strength (right panel) at maximal loading in tripod pinch (pinch gauge) before treatment and during 1 year after surgery, for patients treated with the Artelon CMC spacer (n = 36) or trapezium excision and tendon interposition (n = 26). Dots and error lines show median/mean values and confidence intervals, and the p-values are for change up to 1 year.

In the spacer group, the mean change in tripod pinch strength was 1.1 kg (SD 3.8, p = 0.09) after 1 year and in the control group it was 0.2 kg (SD 2.3, p = 0.7) (Figure 4, right panel), with no statistically significant difference between the groups (p = 0.06) (Table). The mean change in key pinch after 1 year was 0.4 kg (SD 3.5, p = 0.5) in the spacer group and –0.2 kg (SD 3.7, p = 0.8) in the control group. The corresponding figures for change in transverse volar grip strength in the two groups were 3 kg (SD 11, p = 0.1) and 4 kg (SD 9, p = 0.01), respectively. The median VAS values of perceived pain had become significantly reduced after 1 year in both groups (Figure 4, left panel).

The range of motion evaluated as mean radial abduction of the thumb increased during follow-up in the spacer group from 55° (SD 17) to 60° (SD 20) after 1 year (p = 0.02), and mean palmar abduction increased from 54° (SD 17) to 60° (SD 18) after 1 year (p = 0.003). The corresponding figures in the control group were 48° (SD 15) before treatment to 53° (SD 19) after 1 year (p = 0.2) for radial abduction, and 51° (SD 16) before treatment to 52° (SD 16) after 1 year (p = 0.8) for palmar abduction.

**Figure 5.**
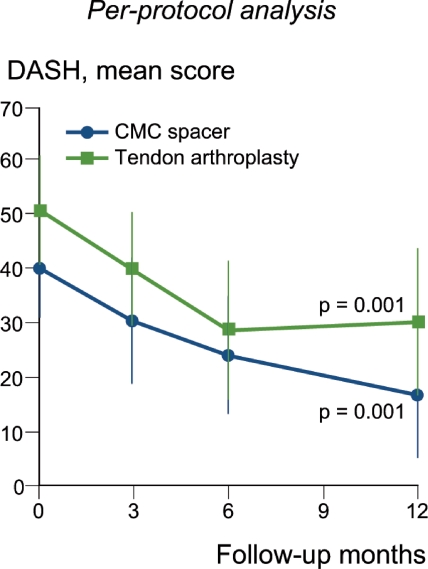
The DASH outcome in patients treated with the Artelon CMC spacer (n = 13) or trapezium excision and tendon interposition (n = 15). The score ranged from 0 (no disability) to 100 (most severe disability). Only patients with surgery in the thumb of the dominant hand are included, due to the character of the questions. Dots and error lines show mean values and confidence intervals, and the p-values are for change up to 1 year.

The mean DASH score improved in both groups (Figure 5). The median decrease in score after 1 year was –26 (–49 to 1) in the spacer group and –18 (-46 to 1) in the control group, including only those with surgery in the dominant thumb. Patient assessments of their thumb function after 1 year revealed a score above 3 in 60% (21/35) of the patients in the spacer group as compared to 65% (17/26) in the control group. The corresponding figures for satisfaction were 66% (23/35) and 69% (18/26), respectively.

## Discussion

We evaluated a new treatment for thumb base OA with insertion of an Artelon CMC spacer and compared it to trapezium excision with tendon interposition arthroplasty. The study had several strengths: it was controlled, randomized, and observer-blinded—i.e. the observers carrying out the follow-up investigations were not informed about which surgical procedure the individual patient obtained—and included a relatively large number of patients treated at 7 clinics.

The new surgical technique with the Artelon CMC spacer, however, involved a challenging factor for the study. 3 techniques for interposition arthroplasty were used in the control group, as it was decided that each investigator should use the technique that he or she was most familiar with. This means that all investigators had long experience of tendon arthroplasty. In contrast, all but one of the investigators had no previous experience of the Artelon CMC spacer. There are a few important differences between the two surgical techniques that may have had an effect on the study. (1) In tendon arthroplasty, a simultaneous OA in the STT joint could be accepted since the trapezial bone is removed. Thus, careful assessment of a potential concomitant OA of the STT joint is not crucial. In contrast, in treatment with the Artelon CMC spacer a thorough pre-treatment evaluation to exclude a combined OA is important in order to achieve full pain relief. (2) Most surgeons do not use antibiotics in connection with tendon arthroplasty, but, as in other surgical procedures with joint implants, the use of antibiotics is recommended in treatment with the Artelon CMC spacer. The lack of preoperative antibiotics appears to be important. 5 of the 6 patients from whom the spacer was removed before 1 year did not receive antibiotics. 2 of them had clinical symptoms of infection, but cultures on these explants were negative. However, delayed infection (defined as a delay of 3–24 months) is characterized by subtle signs and symptoms that are sometimes not even suggestive of infection (Zimmerli 2006), i.e. the diagnosis of prosthetic joint-associated infections is difficult and in many patients the only symptom may be pain (Anguita-Alonso et al. 2005). The infectious agent may be present exclusively as a device-associated biofilm (Zimmerli 2006, Esposito and Leone 2008). (3) Independent evaluation of the postoperative radiographs revealed that the amount of bone removed from the trapezial bone differed. The postoperative appearance on radiographs ranged from a large resection of the trapezium to an almost invisible resection, indicating that the surgical technique differed between the surgeons. In some patients also the opposite, metacarpal, joint surface was incorrectly removed. The importance of training may have been underestimated. The above factors may have been the cause of the high rate of deviations from the protocol in this study.

In a previous pilot study with only 1 clinic involved, pinch strength after Artelon CMC spacer implantation was found to be statistically significantly greater than after tendon interposition arthroplasty (Nilsson et al. 2005). This result could not be confirmed in the present study. To evaluate the influence of protocol deviations, a per-protocol analysis was also carried out, including only patients without STT OA changes and those in the spacer group who received antibiotics. In this analysis, the difference in tripod pinch strength between groups after 1 year was 1.4 kg after adjustment for baseline values (Table). With limited statistical power, however, the difference did not reach statistical significance (p = 0.06). In the power calculation performed before the start of the study, a difference between groups of 1 kg in tripod pinch strength was considered clinically relevant.

Trapezium excision with tendon interposition arthroplasty is considered to be the method of choice by most authors for surgical treatment of thumb base OA. However, many patients experience very long rehabilitation time after surgery and impaired pinch strength. The control group in this study showed better results in terms of complications, pain relief, and pinch strength than those reported in the literature. However, it still seems justified to try to improve the surgical treatment of this single joint disease in which only the CMC joint is affected. Preservation of the length of the first ray including the STT joint is preferable and ought to provide an anatomical base for better thumb function.

Previous attempts to use total joint replacements of the CMC joint have encountered problems with instability and implant dislocation. Regarding the cemented SR trapeziometacarpal prostheses, only 40% (8/19) of patients were found to maintain an excellent or good result 33 months after surgery (Perez-Ubeda et al. 2003). Also, previous efforts to replace the CMC joint with implants made from polymers such as silicone, expanded polytetrafluoroethylene (ePTFE), polypropylene, and collagen have been associated with disadvantages. The use of Silastic trapezial implants is associated with wear, deformation of the implant, and foreign body reactions (Karlsson et al. 1992). Pellegrini and Burton (1986) found a 25% failure rate with silicone implants. Reactions following ePTFE implants have been reported in four-fifths of the patients (Greenberg et al. 1997). Muermans and Coenen (1998) compared Gore-Tex and polypropylene (Marlex) implants with trapezial excision ECRL-tendon interposition. The Marlex implant was well tolerated, but Gore-Tex implants were associated with synovitis, characterized by pain and osteolysis in one-third of the patients. Belcher and Zic (2001) compared trapeziectomy alone with interposition of porcine dermal collagen xenograft (Permacol). The study was terminated prematurely due to adverse reactions.

In the present study, the stability of the CMC-I joint was good in both groups. The range of motion was good, as in the pilot study (Nilsson et al. 2005), in spite of the ligament reinforcement in the spacer procedure producing a firm fixation between the metacarpal base and os trapezium. There was no statistically significant difference between the groups regarding palmar and radial abduction, or regarding retroposition of the thumb. Thus, flattening of the hand was not a problem, supporting the idea that the Artelon CMC spacer permits motion between the metacarpal bone and the implant.

In the total group of spacer patients, including also those with STT changes and lack of antibiotics, a number of patients experienced pain and swelling at the site of implantation. Thus, the reported complications in this group were related to presence of pain, which was the main cause of removal of 6 implants during the 1-year follow-up. In 6 of the other patients who reported pain at 1 year, the CMC spacer has later been removed and a tendon arthroplasty performed. 9 of these 12 patients did not receive antibiotics. 4 of the later 6 implants, explanted after the 1-year follow-up, were available for histological analysis. In 3 cases a general inflammatory reaction was noted in the surrounding soft tissue, but not specifically located at the Artelon material. This is consistent with the results from the 6 first explants. There were no complications of pain in the control group. An overall evaluation of all relevant factors indicated that in 3 cases the symptoms leading to explantation could be due to deviations from the recommended surgical procedure, and in 1 case the preoperative radiograph showed STT OA. Thus, in the latter case pain relief could not be expected from treatment of the CMC-OA only. In 1 case, the implant had loosened from its fixation to the trapezial bone after a heavy lift. The revision procedures included excision of both the implant and the trapezium, followed by prosthesis surgery in 1 case, and conversion to a tendon interposition arthroplasty in 4 cases. All but one of these cases obtained a good result after this procedure, comparable with primary tendon arthroplasty. Adverse tissue reactions around implants could be due to poor fixation of the implant, causing mechanical irritation or a foreign-body reaction to the material. Choung and Tan (2008) have reported a case with pain and swelling in the region of the thumb base after implantation of an Artelon CMC spacer. A biopsy showed signs of inflammation with the presence of multinucleated giant cells consistent with foreign body reaction. The chemical and physical properties of the biomaterials themselves may lead to chronic inflammation, whereas motion at the implant site by the biomaterial may also produce chronic inflammation (Ratner et al. 1996). Thus, the symptoms and the histological results described by Choung and Tan (2008) may be caused by poor fixation of the spacer, indicated by the radiograph showing erosion around the screw heads—which would be consistent with screw movements.

In summary, comparison of a new surgical technique with a well-proven technique involves a challenge, including a clear risk of protocol deviations. The intention-to-treat analysis of tripod pinch strength, the primary outcome measure, did not show any statistically significant superiority of the Artelon CMC spacer over tendon interposition arthroplasty. A thorough patient selection, i.e. exclusion of cases with STT changes and proper use of preoperative antibiotics, appears to be important for the results. Surgery with the Artelon CMC spacer is tissue-preserving, as it spares most of the trapezium and does not require tendon harvest. In addition, the CMC spacer procedure permits the future use of other surgical methods, if required.

**Table:** Pinch and grip strength, and perceived pain at maximal loading

	CMC spacer		Tendon arthroplasty		Difference between groups		
	Pre-treatment	12 months	Pre-treatment	12 months	Change 0–12 months		
	mean (SD)	mean (SD)	mean (SD)	mean (SD)	Adjusted for baseline		
	median (range)	median (range)	median (range)	median (range)	mean	95% CI	p-value
Intention-to-treat	n = 73	n = 63	n = 37	n = 35			
*Strength*							
Tripod pinch (kg)	5.1 (2.4)	5.7 (3.5)	4.5 (2.5)	5.0 (2.1)	0.3	-0.8–1.4	0.6
	5.0 (1.2–15)	5.3 (1–19)	4.0 (1–12)	5.0 (1–9.5)			
Key pinch (kg)	5.7 (2.6)	5.8 (3.0)	5.3 (3.3)	5.2 (2.0)	0.5	-0.6–1.5	0.4
	5.2 (2–14)	5.5 (1.5–19)	4.7 (1–20)	5.0 (2–10)			
Volar grip (kg)	22 (10)	24 (10)	18 (9)	22 (11)	-0.3	-4.1–3.4	0.9
	20 (5–75)	24 (2–57)	17 (1–40)	22 (2–40)			
*Pain*							
Tripod pinch (VAS)	4.4 (2.7)	2.6 (2.7)	5.3 (2.9)	1.3 (2.4)	1.5	0.4–2.5	0.007
		4.3 (0–10)	2.0 (0–10)	5.0 (0–10)	0 (0–9)		
Key pinch (VAS)	4.5 (2.6)	2.6 (2.5)	4.9 (2.6)	1.2 (2.0)	1.4	0.4–2.4	0.005
		4.9 (0–9)	2.0 (0–9)	5.0 (0–10)	0 (0–8)		
Per-protocol	n = 39	n = 36	n = 28	n = 26			
*Strength*							
Tripod pinch (kg)	5.9 (2.7)	7.0 (3.8)	4.9 (2.8)	5.0 (2.2)	1.4	-0.1–3.0	0.06
6.0 (1.2–15)	6.5 (2–19)	4.4 (1–12)	5.0 (1–9.5)				
Key pinch (kg)	6.5 (2.9)	6.9 (3.4)	5.4 (3.6)	5.3 (2.1)	1.4	-0.1–2.8	0.07
		6.0 (2–14)	6.3 (3–19)	4.6 (1–20)	5.0 (2–10)		
Volar grip (kg)	24 (11)	27 (11)	20 (9)	23 (10)	0.6	-4.1–5.4	0.8
		22 (5–75)	27 (2–57)	20 (8–40)	24 (6–40)		
*Pain*							
Tripod pinch (VAS)	3.9 (2.8)	2.2 (2.7)	5.4 (3.1)	1.4 (2.2)	1.0	-0.3–2.4	0.1
		3.3 (0–9)	1.0 (0–10)	5.0 (0–10)	0 (0–7)		
Key pinch (VAS)	3.7 (2.7)	2.2 (2.5)	4.9 (2.8)	1.2 (1.8)	1.1	0–2.3	0.06
		3.9 (0–9)	1.5 (0–9)	5.0 (0–10)	0 (0–5)		
